# Bioengineered Nisin A Derivatives Display Enhanced Activity against Clinical Neonatal Pathogens

**DOI:** 10.3390/antibiotics11111516

**Published:** 2022-10-30

**Authors:** Anna Desmond, Fiona O’Halloran, Lesley Cotter, Colin Hill, Des Field

**Affiliations:** 1 Department of Biological Sciences, Munster Technological University, T12 P928 Cork, Ireland; 2 School of Microbiology, University College Cork, T12 YN60 Cork, Ireland; 3 APC Microbiome Ireland, University College Cork, T12 YN60 Cork, Ireland

**Keywords:** antibacterial peptide, bioengineered peptide, nisin, *Streptococcus agalactiae*, *Staphylococcus capitis*, neonatal infections

## Abstract

Neonatal infection is a significant cause of mortality and morbidity in infants. The global incidence of multi-drug resistance continues to rise among neonatal pathogens, indicating a need for alternative treatment strategies. Nisin is an antimicrobial peptide that exhibits broad-spectrum activity against a wide variety of clinical pathogens and can be used in combination with antibiotics to improve their effectiveness. This study examined the activity of nisin and bioengineered derivatives against multi-drug resistant *Streptococcus agalactiae* and *Staphylococcus capitis* isolates and investigated the potential synergy between nisin peptides and selected antibiotics. Whole genome sequence analysis of the strains revealed the presence of multi-drug resistant determinants, e.g., macrolide, tetracycline, β-lactam, aminoglycoside, while the *S. agalactiae* strains all possessed both *nsr* and *nsr*FP genes and the *S. capitis* strains were found to encode the *nsr* gene alone. Deferred antagonism assays demonstrated that nisin PV had improved antimicrobial activity against all strains tested (*n* = 10). The enhanced specific activity of this peptide was confirmed using minimum inhibitory concentrations (MIC) (0–4-fold lower MIC for nisin PV) and broth-based survival assays. Combinations of nisin peptides with antibiotics were assessed for enhanced antimicrobial activity using growth and time-kill assays and revealed a more effective nisin PV/ampicillin combination against one *S. capitis* strain while a nisin A/erythromycin combination displayed a synergistic effect against one *S. agalactiae* strain. The findings of this study suggest that nisin derivatives alone and in combination with antibiotics have potential as alternative antimicrobial strategies to target neonatal pathogens.

## 1. Introduction

Antibiotic therapy is still the main strategy used to treat bacterial infections, despite the rapid spread and persistence of multi-drug resistant bacteria [1]. Antimicrobial resistance (AMR) is currently responsible for approximately 700,000 deaths annually and is predicted to rise to 10 million deaths per year by 2050 [2]. Of particular concern is the increasing number of multi-drug resistant pathogens causing neonatal infections, which account for approximately 30% of all neonatal sepsis mortality worldwide [3]. Severe bacterial neonatal infections are a significant cause of mortality and morbidity in infants.

Group B Streptococcus (GBS) is a major pathogen associated with neonatal infections [4,5,6] and these infections can be associated with severe long-term complications, such as physical growth retardation and neurological disability [7,8,9]. Despite the extensive use of intrapartum antibiotic prophylaxis (IAP), it is estimated that 30–70% of GBS-colonised mothers give birth to GBS-colonised new-borns, of which 1–2% develop early onset infections [10,11]. Importantly, GBS remain the leading cause of neonatal sepsis and meningitis [12,13]. While studies have shown that IAP reduces infection rates, its increased use has raised concerns about the emergence and persistence of AMR among GBS strains, as well as contributing to increased AMR in other common neonatal pathogens [14,15,16]. The most common treatment for infections caused by these pathogens are antibiotics including penicillin, vancomycin, ampicillin, gentamicin, erythromycin, and tetracycline [17,18,19,20]. Unfortunately, many neonatal-associated strains are becoming resistant to these antibiotics with several reports revealing reduced sensitivity or resistance [18,19,20,21]. For example, Doenhardt et al. and Gao et al. have reported that GBS has developed resistance to common antibiotics used to treat neonatal infections, including erythromycin (70.9%), clindamycin (64%) and tetracycline (88.4%) [18,19]. *Staphylococcus capitis* (*S. capitis*), a coagulase negative staphylococcus, has recently emerged as a microbe of concern in neonatal intensive care units (NICUs) in particular, the NRCS-A clone [22]. Worryingly, this strain is resistant to the action of both antibiotics and antiseptics [20]. Rasigade et al. reported that *S. capitis* NRCS-A strains isolated from NICUs were highly resistant to first line antibiotics, including penicillin (99%), methicillin (95.6%) and gentamicin (95.1%) [21]. This clone was also capable of responding to selective pressure from vancomycin exposure, resulting in the fast and stable development of vancomycin resistance [23]. 

The significance of severe neonatal infections coupled with the emergence of antibiotic resistance in pathogenic bacteria and the decline in discovery and development of new, effective antibiotics means that the exploration and development of alternative strategies and novel alternative antimicrobial compounds to conventional therapy are required to treat multi-drug resistant bacterial infections and/or improve the efficacy of existing antibiotics. Alternatives to conventional antibiotic treatment include the use of bacteriophage, antimicrobial peptides, bacteriocins, probiotics and antibodies [24,25,26,27]. 

**Figure 1:**
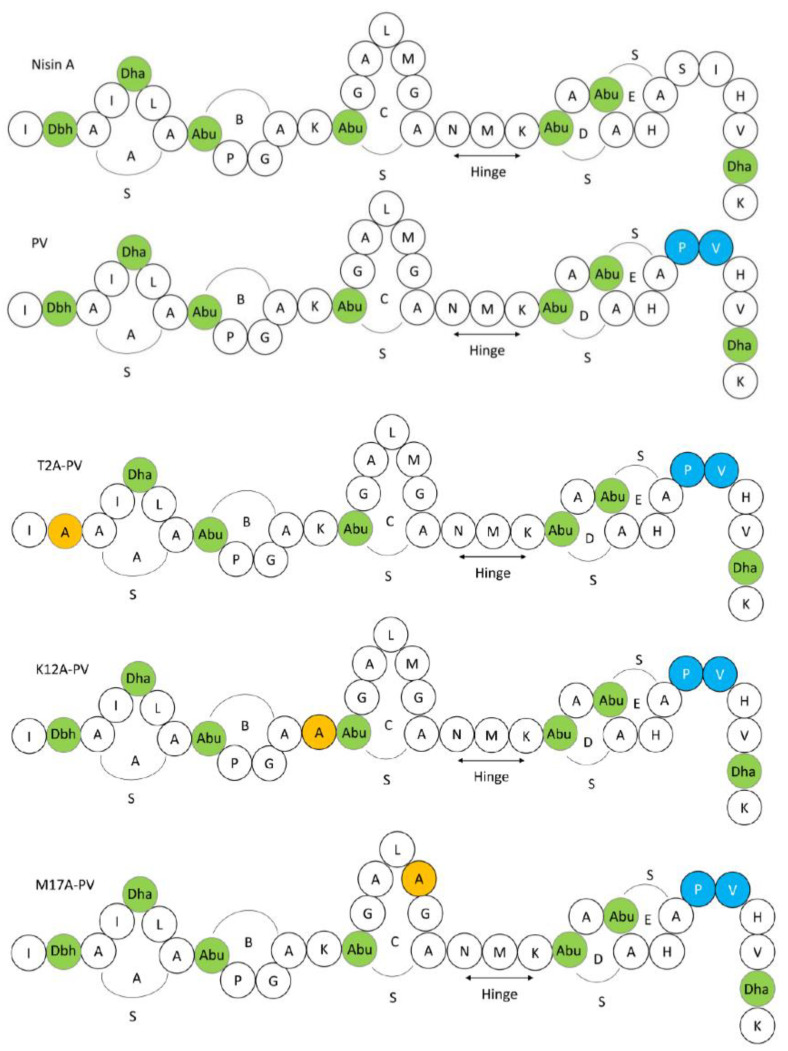
Schematic structures of the 34 amino acid (AA) pentacyclic peptide nisin A and nisin derivatives used in this study, highlighting the position of the five lanthionine rings (A, B, C, D, E) and the hinge region. Residues are represented in the single letter code and post-translational modified AA are indicated in green as follows: Dha: dehydroalanine, Dhb: dehydrobutyrine and Abu: 2-aminobutyric acid. Nisin PV is indicated in blue. Substitute amino acids are indicated in orange.

Nisin, a 34 amino acid pentacyclic peptide (Figure 1), is naturally produced by *Lactococcus lactis*, and exhibits antimicrobial activity against several Gram-positive and Gram-negative bacteria [28,29,30]. Nisin is approved as a natural food preservative and is considered safe for use by the US Food and Drug Administration (FDA) [28]. Several studies have shown that nisin and nisin variants have efficacy against clinical pathogens, including multi-drug resistant organisms [31,32,33]. Notably, variants nisin T (K22T) and nisin PV, were reported to have increased activity against clinical *S. agalactiae* isolates [29,31], while other derivatives, such as nisin HTK, nisin M17Q and nisin T2L have demonstrated increased potency against *Staphylococcus aureus* strains [34]. Some nisin variants have both anti-biofilm and antimicrobial activity against clinical isolates of *Staphylococcus pseudintermedius* and *Streptococcus uberis* [32,33,34,35].

While resistance to nisin is not commonly reported, some pathogens exhibit resistance through mechanisms that include cell wall modifications and biofilm formation [36], as well as the production of nisin specific protein defence mechanisms [37]. Some bacteria, including GBS, possess a nisin resistance gene cluster (nsr) which encodes a two-component signalling system (NsrRK) and two protein systems, the nisin resistance protein (Nsr) and an ABC transporter (NsrFP) [38]. Both NSR and NsrFP work cooperatively to obtain full resistance against nisin. NSR degrades nisin by cleaving the peptide bond between MeLan 28 and Ser 29 at the C-terminal end of the peptide which is mediated by a conserved tail-specific protease domain [31]. This truncated nisin structure (nisin 1–28) exhibits significantly less capacity for pore formation and has up to 100-fold decreased antimicrobial activity [36,37,38,39]. Importantly, nisin is gene-encoded and thus can be modified to generate novel derivatives with a view to enhancing bioactivity [28]. Over the last decade, several bioengineered nisin derivatives have been generated that exhibit increased antimicrobial activity, increased peptide stability, improved pharmacokinetic properties [28,29,40] and reduced susceptibility to nisin degrading proteins [41]. 

With increasing concerns about antibiotic resistance, exploring alternative therapeutic strategies is critical. However, reports to date emphasize that some of these approaches can be associated with strain-specific effects, rather than -broad-spectrum activity [27]. One potential solution is combination therapy, where two or more antimicrobials with differing modes of action function in synergistic fashion. Such action has the potential to restore the activity of antibiotics [42] and reduce the concentrations of antibiotics needed to treat infections [43], thus diminishing the risk of drug toxicity [44]. Furthermore, bacteria are less likely to overcome the action of two distinct antimicrobials, and in that way may lessen further the capacity for bacterial antibiotic resistance development. [45]. However, several factors need to be considered with antimicrobial combination approaches, including knowledge of their mode of action, dose regimen, potential mechanisms of resistance and drug metabolism [44,46]. 

Nisin and antibiotics have different modes of action, making them suitable candidates for combination therapy. Nisin inhibits bacterial cell wall synthesis by binding to lipid II, which then enhances its ability to form pores in the cell membrane leading to the loss of metabolites and cell death [28]. Several studies have shown that combining nisin with antibiotics can increase the antibacterial activity against a range of pathogens, such as *E. faecalis*, *S. aureus*, *S. agalactiae* and *S. pseudintermedius* [31,32,45]. In a recent study by El-Kazzas and El-Khier the combination of nisin and chloramphenicol displayed synergistic action against 82.6% of VRE isolates, and against the remaining isolates (17.4%) an additive effect was observed [47]. Similarly, Tong et al. noted that nisin in combination with chloramphenicol or penicillin had synergistic effects against *E. faecalis* [45]. The nisin derivative I4V in combination with penicillin demonstrated the ability to completely diminish the growth of *S. pseudintermedius*, and a nisin I4V and chloramphenicol combination proved effective against biofilms of *S. pseudintermedius* [32]

Thomas et al. reported that the combination of nisin A and polymyxin B acted synergistically against 38% of drug-resistant and 86% of pandrug-resistant *Acinetobacter baumannii* isolates [48]. Notably, Castelani and coworkers demonstrated partial synergy between nisin and the synthetic cationic lipid dioctadecyldimethylammonium bromide against two *S. capitis* strains as part of a larger study involving antibiotic-resistant Staphylococcus spp. isolated from bovine mastitis [49]. These studies support the potential for antimicrobial strategies that include nisin or its derivatives, or combinations with antibiotics that can improve their effectiveness against clinically relevant strains.

In this study, a bioengineered nisin derivative previously shown to be resistant to the action of NSR [41] was used as template to generate three banks of bioengineered nisin PV derivatives (T2X-PV, K12X-PV, M17X-PV) in a bid to identify novel peptides that exhibit enhanced efficacy against a selection of neonatal-associated strains of *S. agalactiae* and *S. capitis*. In addition, combinations of nisin and two derivatives (nisin PV and nisin K12A-PV) were assessed against *S. agalactiae* and *S. capitis* isolates in combination with several antibiotics to identify potential synergistic combinations as a treatment option for these neonatal pathogens.

## 2. Results

### 2.1. Bioinformatic and Genomic Analysis 

The genomes of *S. agalactiae* (*n* = 5) and *S. capitis* isolates (*n* = 5) were investigated for (i) nisin resistance and (ii) antibiotic resistance determinants using ResFinder 4.1. The genomic DNA of 100% (*S. agalactiae n* = 5; *S. capitis n* = 5) of isolates were shown to encode for the nisin resistance protein Nsr and only 50% of isolates (*S. agalactiae n* = 5) encoded for NsrFP. Results are shown in Table 1. 

Several macrolide resistance determinants were found including *erm*A, *erm*B, *erm*C, *msr*D, *mef*A, *mre*A and *isa*C and a number of strains possessed more than one of these resistance genes. The *mre*A gene was found in 50% of isolates, while only 10% of isolates possessed *erm*C (Table 1). Additionally, 40% of isolates possessed *tet*M and 20% isolates possessed *tet*O, which encodes tetracycline resistance.

The *bla*Z (associated with penicillin resistance) was found to reside in 50% of strains and 40% were also found to harbour both the *mec*A gene and the *acc*(6′)-*aph*(2″) gene (associated with aminoglycoside resistance). Additionally, 30% of isolates possessed *fus*B that encodes for fusidic acid resistance. Among all the isolates tested, at least one resistance gene was found to reside in at least one of the strains, e.g., 10% of strains harboured *fos*D (associated with fosfomycin) and *isa*C (associated with macrolide resistance). Interestingly, 10% of strains contained resistance genes associated with quaternary ammonium compounds which are commonly found in disinfectants.

### 2.2. Screening Nisin Derivatives for Enhanced Antimicrobial Activity

**Figure 2:**
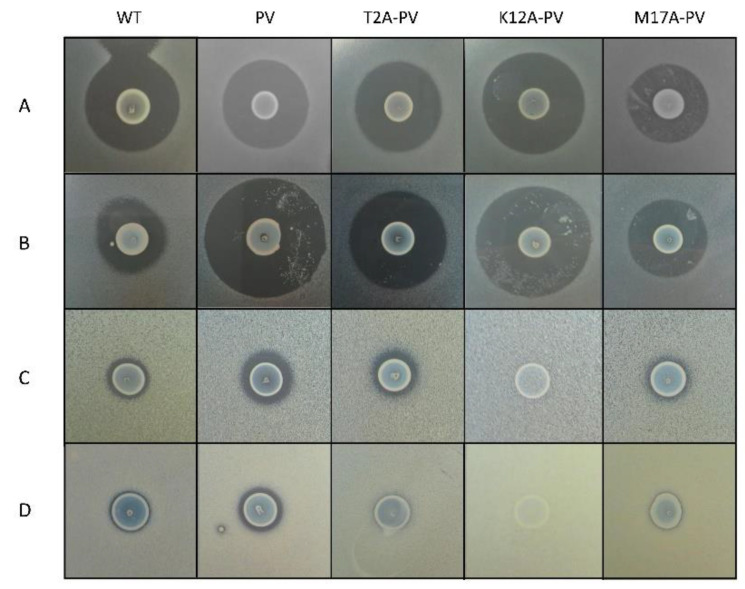
Deferred antagonism assay illustrating the zones of inhibition produced by the wild-type nisin A (WT), nisin PV and its derivatives nisin T2A-PV, nisin K12A-PV and nisin M17A-PV against (**A**) *L. lactis* MG1614 (**B**) *L. lactis* MG1614 pNP40 (**C**) *S. agalactiae* CIT 67 (**D**) *S. capitis* AV80. All plates were supplemented with sub-inhibitory concentration (10 µg/L) of nisin in the form of nisaplin to ensure induction of the nisin mutants.

Site-saturation mutagenesis [28] was employed to generate bioengineered nisin derivatives using the plasmid pCI372-nisinA-PV as template. Deferred antagonism agar-based assays were employed to assess the bioactivity (i.e., the combined impact on production and activity) of the nisin derivatives (PV, K12X-PV, T2X-PV, and M17X-PV where X = any amino acid) produced by each strain. The combined banks of ~450 independent producers were screened to identify those which displayed greater bioactivity than the wild type nisin A peptide and the derivative nisin PV against *L. lactis* MG1614 and *L. lactis* MG1614 pNP40, which expresses the nisin resistance protein and against clinical isolates of *S. agalactiae* (*n* = 5) and *S. capitis* (*n* = 5). Although several derivatives at each location were identified including T2M-PV, T2S-PV, T2G-PV, T2A-PV, T2F-PV, T2P-PV, T2Q-PV, T2I-PV, T2L-PV, T2K-PV, K12D-PV, K12Y-PV, K12T-PV, K12S-PV, K12R-PV, K12F-PV, K12C-PV, K12N-PV, K12H-PV, K12L-PV, K12I-PV and M17Q-PV, most of those did not exhibit improved activity in deferred antagonism assays. However, it was apparent that three variants had superior bioactivity against the *L. lactis* strains compared to nisin A and one, was superior to nisin PV against *L. lactis* MG1614 (Figure 2). The three variants (one from each bank) were carried forward against the entire collection of neonatal-associated clinical isolates (Figure 2). 

**Figure 3:**
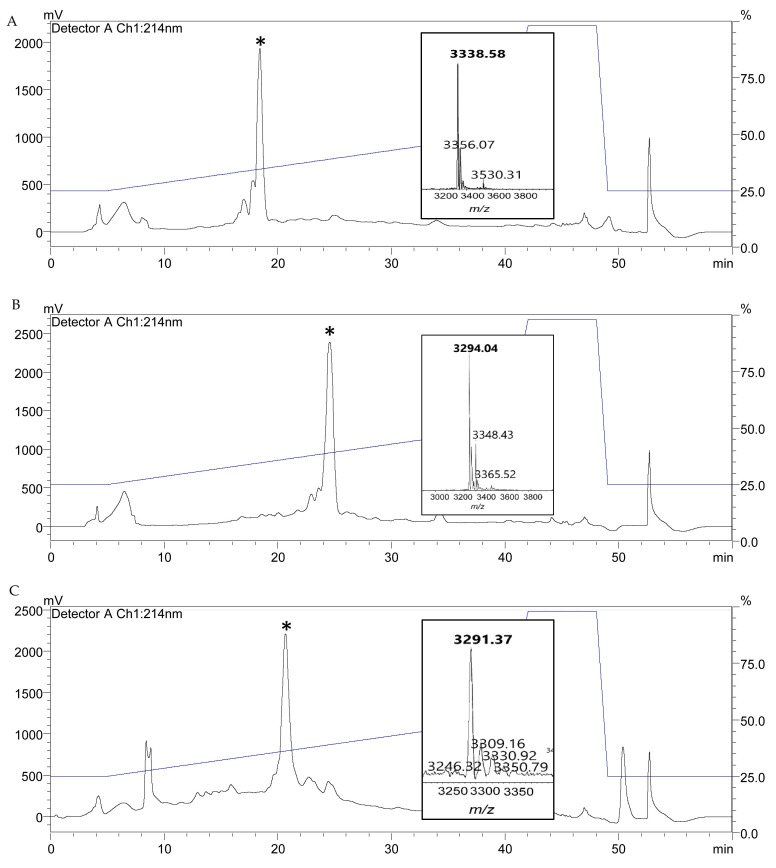
RP-HPLC profiles of (**A**) T2A-PV, (**B**) K12A-PV and (**C**) M17A-PV. Purified powder from RP-HPLC fractions were subjected to MALDI-ToF mass spectrometric (MS) analysis (inset) to confirm the expected mass of T2A-PV (3338 Da), K12A-PV (3294 Da) and M17A-PV (3291 Da).

Following screening, wild type nisin A, nisin PV and the three new variants were further analyzed by colony mass spectrometric (CMS) analysis. CMS analysis confirmed that a peptide mass of 3338 Da corresponded to nisin T2A-PV (threonine at position 2 altered to alanine), a mass of 3294 Da corresponded to nisin K12A-PV (lysine at position 12 altered to alanine) and a mass of 3291 Da corresponded to nisin M17A-PV (methionine at position 17 altered to alanine) (AA positions highlighted in Figure 1). Notably, the parental strain producing nisin PV [41] displayed the greatest bioactivity against the majority of pathogenic indicators (Figure 2). Although the bioactivity of the nisinT2A-PV, nisin K12A-PV and nisin M17A-PV producing strains was enhanced against the lactococcal targets compared to wild-type and PV, this was not apparent against the pathogenic isolates (Figure 2). However, all peptide derivatives including nisin PV, T2A-PV, K12A-PV, and M17A-PV were purified by HPLC for specific activity determination given that the impact on production of these peptides was as yet unknown. Purified peptides were subjected to MALDI-TOF mass spectrometry to confirm the correct mass in each case (Figure 3).

### 2.3. MIC-Based Investigations of the Specific Activity of Nisin Derivatives

The specific activity of nisin PV, nisin T2A-PV, nisin K12A-PV and nisin M17A-PV were assessed and compared to nisin A using broth-based minimum inhibitory concentration (MIC) determination assays against *L. lactis* MG1614, *L. lactis* MG1614 pNP40 as well as five *S. agalactiae* (Nsr+, NsrFP+) and five *S. capitis* (Nsr+) isolates (Table 2). MICs were recorded as the lowest concentration of peptide that resulted in the absence of visible growth after 16h at 30 °C or 37 °C. When *L. lactis* MG1614 was evaluated, the derivatives nisin T2A-PV, nisin K12A-PV and nisin M17A-PV proved to be less active than the wild type nisin A (0.02 µg/mL) exhibiting MIC values of 0.26, 0.13 and 0.56 µg/mL, respectively, (Table 2). However, when *L. lactis* pNP40 (Nsr+) was used as the target, all three derivatives displayed enhanced activity compared to nisin A (0.13, 0.06 and 0.06 µg/mL, respectively, though these values were between 2 and 4 fold less than that obtained for the nisin PV derivative (0.03 µg/mL). MIC values for nisin A against *S. agalactiae* strains ranged from 0.78 to 12.5 µg/mL compared to the MIC values for nisin PV which ranged from 0.38 to 12.5 µg/mL. For four of the five *S. agalactiae* strains nisin PV displayed enhanced activity, with a 2-fold increase in potency compared to the activity of nisin A (Table 2). Interestingly, for one *S. agalactiae* isolate (CIT 85) the potency of nisin PV decreased 2-fold compared to nisin A. Results obtained for the nisin K12A-PV peptide demonstrated that the bioactivity of this derivative against the *S. agalactiae* isolates was also strain dependent, with MIC values ranging from 1.56 to 25 ug/mL, and the derivative was not more active than the wild-type peptide, nisin A. Indeed, the potency of nisin K12A-PV decreased 2-fold against three *S. agalactiae* strains (CIT 87, CIT 364, and CIT 239) compared to nisin A (Table 2). Notably, when comparing the activity of derivatives PV and K12A-PV, the latter was less potent, with a 4-fold decrease in potency against *S. agalactiae* isolates CIT 87, CIT 239, and CIT 364 and a 2-fold decrease in activity against *S. agalactiae* CIT 67 compared to nisin PV. Results obtained for nisin T2A-PV demonstrated that the specific activity of this derivative against *S. agalactiae* CIT 67 decreased by 2 and 4-fold compared to nisin A and nisin PV respectively. Similarly, nisin M17A-PV displayed a 4 and 8-fold decrease in potency against *S. agalactiae* CIT 67 compared to nisin A and nisin PV (Table 2). 

When the activity of nisin A and nisin derivatives were analyzed against *S. capitis* isolates, MIC values for nisin A against *S. capitis* strains ranged from 12.5 to 50 µg/mL compared to values for nisin PV which ranged from 6 to 50 µg/mL (Table 2). Nisin PV was the only derivative to exhibit enhanced specific activity against two of the five isolates, with a 2-fold and 4-fold increase in potency against *S. capitis* BA06 and *S. capitis* AY41, respectively, compared to nisin A (Table 2). The MIC values for the nisin derivative K12A-PV against the *S. capitis* isolates ranged from 50 to 100 µg/mL and the derivative was less active compared nisin A and nisin PV (Table 2). Notably, T2A-PV displayed a 2-fold increase in activity against *S. capitis* AY41 compared to nisin A, however, it also displayed 2-fold decrease in potency compared to nisin PV. Similarly, nisin M17A-PV demonstrated a 2-fold increase in potency against *S. capitis* AY41 compared to nisin A as well as 2-fold decrease in activity when compared to nisin PV (Table 2).

### 2.4. MIC Investigations of Antibiotics

To establish suitable concentrations for combination studies with nisin peptides, the MICs for a range of antibiotics were determined. MICs for penicillin, ampicillin, gentamicin, erythromycin, and vancomycin were established against all *S. agalactiae* and *S. capitis* strains (Table 2). High MICs ranging from 0.78 to 3.13 µg/mL were recorded for erythromycin against four *S. agalactiae* isolates. Similarly, MICs ranging from 0.78 to 1.56 µg/mL were recorded for ampicillin against three *S. agalactiae* strains and MICs of 6.25 and 25 µg/mL were recorded with gentamicin against four *S. agalactiae* isolates (Table 2). In contrast, all *S. agalactiae* strains were sensitive to penicillin ranging from 1.56–0.097 µg/mL.

For four of the five *S. capitis* isolates, high MIC values were recorded with ampicillin, ranging from 6.25 to >12.5 µg/mL (Table 2). Similarly, MIC values of 6.25 to >12.5 µg/mL were recorded for penicillin against four *S. capitis* strains. When gentamycin was used, four *S. capitis* strains displayed a MIC value >50 µg/mL, and three strains exhibited resistance to erythromycin (Table 2). Both *S. capitis* AV80 and BD01 strains demonstrated resistance (3.13 µg/mL) to vancomycin, while *S. capitis* strains AR18 and BA06 showed heteroresistance (1.56 µg/mL) to vancomycin. (Table 2). *S. capitis* AY41 proved to be susceptible to most of the antibiotics utilized in this study. These data highlight the drug resistant nature of these *S. agalactiae* and *S. capitis* isolates and are in agreement to what has been previously reported [2,3].

### 2.5. Growth Curve Analysis of the Activity of Nisin A and PV Alone and in Combination with Antibiotics

**Figure 4:**
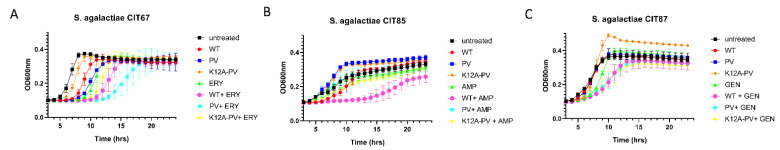
Impact of combinations of nisin derivatives and antibiotics on growth of representative *Streptococcus agalactiae* isolates. Concentrations used were established from MIC evaluation. Growth curve analysis of (**A**) *S. agalactiae* CIT 67 in untreated (black square), 1 µg/mL of nisin A (red circle), 1 µg/mL of nisin PV (blue circle), 1 µg/mL of K12A-PV (orange diamond), 0.33 µg/mL of erythromycin (green triangle), and the combinations of nisin A and erythromycin (pink circle), PV and erythromycin (light blue square), K12A-PV and erythromycin (yellow diamond), (**B**) *S. agalactiae* CIT 85 in untreated (black square), 1.5 µg/mL of nisin A (red circle), 1.5 µg/mL of nisin PV (blue circle), 1.5 µg/mL of K12A-PV (orange diamond), 0.024 µg/mL of ampicillin (green triangle), and the combinations of nisin A and ampicillin (pink circle), PV and ampicillin (light blue square), K12A-PV and ampicillin (yellow diamond) and (**C**) *S. agalactiae* CIT 87 in untreated (black square), 0.26 µg/mL of nisin A (red circle), 0.26 µg/mL of nisin PV (blue circle), 0.26 µg/mL of K12A-PV (orange diamond), 8.3 µg/mL of gentamicin (green triangle), and the combinations of nisin A and gentamicin (pink circle), PV and gentamicin (light blue square), K12A-PV and gentamicin (yellow diamond). The means and standard deviations of the three independent determinations are presented.

Having demonstrated the increased specific activity of the nisin derivatives against specific *S. agalactiae* strains, the impact of sub-lethal concentrations of the purified peptides alone and in combination with different antibiotics was investigated (Figure 4). Data showed that the antimicrobial activity of the combinations was strain dependent in that some nisin derivative/antibiotic combinations performed better than wild type nisin/antibiotic combinations. The combination of nisin PV (1 μg/mL, 1/3 MIC) with erythromycin (0.33 μg/mL, 1/3 MIC) against isolate *S. agalactiae* CIT 67 caused a significant delay (*p* < 0.05) in growth compared to the untreated control, individual agents alone and other combinations (Figure 4A). Notably, the combination of nisin A (1.5 μg/mL, 1/4 MIC) with ampicillin (0.024 μg/mL, 1/4 MIC) significantly reduced (*p* < 0.05) bacterial growth compared to the combination of nisin PV with ampicillin against *S. agalactiae* CIT 85 (Figure 4B). Both wild type/gentamycin and PV/gentamycin combinations displayed similar activity to the untreated control and individual agents alone against CIT 87. Results observed for the nisin K12A-PV peptide demonstrated that it was not more active either alone or in combination compared to the wild-type peptide, nisin A or nisin PV. 

**Figure 5:**
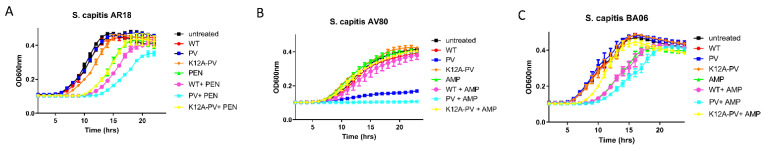
Impact of combinations of nisin derivatives and antibiotics on growth of representative *Staphylococcus capitis* isolates. Concentrations used were established from MIC evaluation. Growth curve analysis of (**A**) *S. capitis* AR18 in untreated (black square), 6.25 µg/mL of nisin A (red circle), 6.25 µg/mL of nisin PV (blue circle), 6.25 µg/mL of K12A-PV (orange diamond), 6.25 µg/mL of penicillin (green triangle), and the combinations of nisin A and penicillin (yellow diamond), PV and penicillin (light blue square), K12A-PV and penicillin (yellow diamond), (**B**) *S. capitis* AV80 in untreated (black square), 12.5 µg/mL of nisin A (red circle), 12.5 µg/mL of nisin PV (blue circle), 12.5 µg/mL of K12A-PV (orange diamond), 6.25 µg/mL of ampicillin (green triangle), and the combinations of nisin A and ampicillin (pink circle), PV and ampicillin (light blue square), K12A-PV and ampicillin (outlined square) and (**C**) *S. capitis* BA06 in untreated (black square), 3.1 µg/mL of nisin A (red circle), 3.1 µg/mL of nisin PV (blue circle), 3.1 µg/mL of K12A-PV (orange diamond), 6.25 µg/mL of ampicillin (green triangle), and the combinations of nisin A and ampicillin (pink circle), PV and ampicillin (light blue square), K12A-PV and ampicillin (yellow diamond). The means and standard deviations of the three independent determinations are presented.

Three *S. capitis* strains were selected for studies with nisin peptides alone and in combination with antibiotics. *S. capitis* AV80 was the most susceptible strain to nisin PV alone and in combination compared to the other strains tested. Nisin PV when applied at a concentration of 12.5 μg/mL (1/4 MIC) caused a significant delay in the growth of *S. capitis* AV80 (~10 h) (Figure 5B). The enhanced potency of nisin PV with ampicillin against *S. capitis* AV80 was evident, with complete inhibition of the isolate over 24 h (*p* < 0.05) (Figure 5B). For isolate AR18, 6.25 μg/mL (1/4 MIC) of penicillin extended the lag phase over 10 h. However, the combinations of nisin variants with penicillin failed to show any difference compared to penicillin alone (Figure 5A). Similarly, the combinations of nisin peptides with ampicillin, did not show enhanced activity compared to ampicillin alone against *S. capitis* BA06 (Figure 5C).

### 2.6. Kill Curve Analysis of the Activity of Peptide Activity Alone and in Combinations

**Figure 6:**
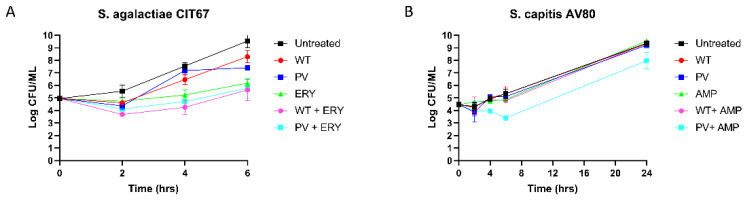
Time-Kill curve analysis of (**A**) *S. agalactiae* CIT 67 *S. capitis* in untreated (black square), 1.5 µg/mL of nisin A (WT) (red circle), 1.5 µg/mL of nisin PV (blue circle), 0.5 µg/mL of erythromycin (green triangle), and combinations of nisin A and erythromycin (pink circle), and nisin PV and erythromycin (light blue square) and (**B**) *S. capitis* AV80 in untreated (black square), 12.5 µg/mL of nisin A (red circle), 12.5 µg/mL of nisin PV (blue square), 6.25 µg/mL of ampicillin (green triangle), and the combinations of nisin A and ampicillin (pink circle), PV and ampicillin (light blue square). The means and standard deviations of three independent determinations are presented.

The bactericidal activity of nisin A and nisin PV combined with erythromycin or ampicillin over a defined period against one *S. agalactiae* and one *S. capitis* strain was investigated using kill curve analysis. *S. agalactiae* CIT67 was exposed to either nisin A or PV at 1.5 µg/mL (1/2 MIC) combined with erythromycin at 0.5 µg/mL (1/2 MIC) over a period of 6 h (Figure 6A). After 2 h, a 2-log reduction in cell numbers was observed for the nisin A or nisin PV peptides with erythromycin compared to the untreated control. When comparing the activity of the nisin A and erythromycin combination to either antimicrobial alone, a 2-log reduction in growth was observed over 4 h, however, re-growth was observed after 6 h (Figure 6A). 

Next, *S. capitis* AV80 was investigated using nisin A or nisin PV at 12.5 µg/mL (1/4 MIC) and ampicillin at 3.75 µg/mL (1/4 MIC), as well as combinations at the same concentrations (Figure 6B). The single treatments of nisin A, nisin PV and ampicillin inhibited bacterial growth over 4 h, however, subsequent re-growth was observed after 6 h. Notably, after 4 h and continuing up to 24 h, a 1-log reduction in cell numbers was evident for the combination of nisin PV with ampicillin, compared to either antimicrobial alone. (Figure 6B).

## 3. Discussion

Group B Streptococci and *S. capitis* are opportunistic pathogens involved in neonatal infections and can be associated with high mortality and morbidity rates [4,5,6]. In this study, *S. agalactiae* and *S. capitis* isolates were examined for antibiotic resistance and nisin resistance determinants. Notably, all of the isolates utilised in the study harboured resistance genes against antibiotics from different classes such as beta-lactams, aminoglycosides, macrolides, and glycopeptides which are the antibiotics of choice in treating neonatal infections [7,8,9]. In the case of the *S. capitis* isolates, the antibiotic resistance profile was in agreement with the study by Butin and coworkers [23]. Notably, this resistance profile is largely consistent with the preferential use of these antimicrobials in NICUs [23] and demonstrate how the broad use of antibiotics can frequently cause initially drug-sensitive commensal bacteria to evolve into multidrug-resistant clones that are capable of worldwide dissemination [50]. Similarly, the resistance of GBS isolates to different antibiotic classes observed in this study is concerning and reveals that alternative treatment strategies are urgently required. The proportions of GBS isolates exhibiting in vitro resistance to erythromycin and clindamycin have increased over the last number of decades [51] Notably, erythromycin and clindamycin are the second-choice antibiotics for GBS infections in cases where penicillin is contraindicated due to allergy [52]. Moreover, resistance to other classes of antibiotics (fluoroquinolone and tetracycline) continues to increase, thereby potentially hindering the effective control of GBS-related neo-natal infections. 

Due to its mode of action and broad range of antimicrobial activity, nisin has attracted attention as a novel therapeutic for the treatment of bacterial infections and more recently for cancer therapy [53]. The ability to alter the peptide sequence through bioengineering provides a means to generate novel derivatives with improved bioactivity. In this study, four nisin derivatives were investigated for enhanced antimicrobial activity against neonatal-associated isolates of *S. agalactiae* and *S. capitis* that are known to possess the nisin resistance protein NSR. Three positions within nisin (T2, K12 and M17) were targeted for mutagenesis based on previous studies highlighting the importance of these positions with respect to enhancing the specific activity of nisin against several pathogens [13,14]. Directed site-saturation mutagenesis of the plasmid harbouring the nisin PV gene that expresses a peptide variant previously shown to be impervious to the action of NSR [41] was carried out. Subsequently, an extensive screen of three bioengineered banks was undertaken and identified three combinatorial derivatives, one at each location (nisin T2A-PV, nisin K12A-PV and nisin M17A-PV) that exhibited enhanced bioactivity. One variant, nisin K12A-PV exhibited improved activity compared to nisin A and nisin PV against a nisin-sensitive lactococcal strain in deferred antagonism assays. In contrast, the three derivatives did not prove to be more bioactive compared to the parental nisin PV against the same lactococcal strain expressing NSR. Despite this, the variants did demonstrate improved potency compared to nisin A when assessed by specific activity tests. Several factors can influence the size and appearance of inhibitory zones in deferred antagonism assays, including peptide solubility, ability to diffuse through the agar and the presence of resistance mechanisms. These factors may explain the results observed between the two assays. Notably, derivatives at position threonine 2 (T2L) and methionine 17 (M17Q) have been previously shown to improve the antimicrobial activity of nisin against pathogenic strains. For example, nisin M17Q displayed enhanced bioactivity compared to nisin A against clinical strains of *S. epidermidis* [54] while nisin T2L was enhanced when compared with nisin A against *S. aureus* [34]. The aim of this study was to generate novel derivatives with improved activity against neonatal-associated *S. agalactiae* and *S. capitis* strains that are known to possess nisin resistance determinants. Against this collection, nisin PV was significantly more effective than nisin A, which is in agreement with a previous study involving GBS strains [31]. Moreover, investigations carried out with purified peptides revealed that nisin PV was 2–4-fold more active compared to nisin A against some strains of *S. capitis*. To our knowledge, this is the first study to investigate the activity of nisin A and nisin derivatives against neonatal isolates of *S. capitis* and is the first description of a bioengineered nisin derivative with improved activity against *S. capitis*, highlighting the benefits of bioengineering to improve the antimicrobial action of nisin against this drug resistant pathogen. 

The introduction of mutations into the nisin PV peptide that alone have been shown to be beneficial when targeting other pathogens including methicillin resistant *S. aureus* (MRSA) [33,34], did not increase the specific activity of nisin PV, indicating that structural considerations are also a factor with regard to the antimicrobial action of nisin. This is borne out by the fact that nisin T2A-PV, nisin K12A-PV and nisin M17A-PV were either equally or less active than parental nisin PV against all of the isolates tested, though still remained twice as active compared to the wild type nisin A peptide. Overall, the contrasting sensitivity of *S. agalactiae* and *S. capitis* to nisin T2A-PV, nisin K12A-PV and nisin M17A-PV provides additional data that some bioengineered nisin derivatives exhibit target-specific variations in potency.

It is worth noting that while the *S. capitis* NCRS-A clone has previously been shown to possess NSR [18], our bioinformatics analysis failed to identify an ABC transporter, NsrFP which is commonly associated with NSR [38] Indeed, given that nisin PV was shown to resist proteolytic cleavage by NSR, it was surprising that some strains (40%) showed equal or reduced susceptibility to nisin PV compared to nisin A and suggests the presence of other transcriptional regulators and two-component systems that act as a source of environmental monitoring and resistance mechanisms. Indeed, some strains of *S. capitis* NCRS-A have been shown to encode an additional cell-wall teichoic-acids-associated cluster (*tar*FIJL) that is important in biofilm formation, attachment to biomaterials, and for protection against cell damage (e.g., glycopeptide resistance) [55].

Combination therapy is an attractive alternative strategy to treat antibiotic resistant strains, particularly, if the approach involves two or more known antimicrobial compounds that have different modes of action and work synergistically to potentially increase the therapeutic efficacy of clinically used antibiotics and reduce the concentrations required for single treatments [42]. Several studies have shown that the addition of nisin can increase the antibacterial activity of antibiotics against a range of clinical pathogens [32,45,47,56]. Antibiotic-nisin combination therapy has the potential to be an effective antimicrobial treatment option for neonatal infections, while also decreasing the development of drug resistance in bacteria [32,45]. Despite the many reports of promising synergistic interactions between bacteriocins and other stressors against clinically relevant bacteria [57], there are little to no reports concerning the combined effects of antibiotic combinations against multi-drug resistant *S. capitis*. Here, we investigated the potential of nisin and two bioengineered nisin derivatives (nisin PV and nisin K12A-PV) alone and in combination with a selection of conventional antibiotics and demonstrate enhanced inhibitory relationships between nisin + ampicillin and nisin + erythromycin. A significantly greater inhibitory effect was observed for nisin PV and ampicillin against one isolate, *S. capitis* AV80. It is worth noting that this isolate was one of the most resistant to both nisin PV (50 µg/mL) and ampicillin (>12.5 µg/mL). Similarly, the combination of nisin A and ampicillin demonstrated enhanced potency against *S. agalactiae* CIT 85, while nisin PV and erythromycin proved a highly efficacious combination against *S. agalactiae* CIT 67, a strain that harbours the *msr*(D), *mre*(A), *mef*(A) genes for erythromycin resistance.

The findings of this study suggest that antibiotic-nisin combination therapy has significant potential as a more effective antimicrobial treatment option for neonatal infections. Although nisin has not yet been commercially approved as a clinical antimicrobial [28], its potential with respect to clinical applications is supported by numerous studies highlighting its activity against human pathogens, including multi-drug resistant strains. Furthermore, this study adds to the growing catalogue of laboratory-based experiments underscoring the application of nisin in conjunction with other conventionally used antibiotics in a bid to restore sensitivity and reduce their minimum effective dose. However, if nisin is to be employed in the biomedical industry, further research is needed to monitor nisin resistance in pathogenic micro-organisms and to investigate the prospect of dedicated nisin resistance determinant (e.g., *nsr* and *nsr*FP) transfer from non-pathogenic bacteria to other clinically pathogenic organisms. Furthermore, the potential to improve the specific activity of nisin through bioengineering opens new opportunities for research into synergy between bioengineered nisin derivatives and antibiotics. However, it should be emphasized that effective synergistic relations between bacteriocins and other antimicrobials in vitro may not inevitably translate to clinical efficacy. Nonetheless, careful optimization of factors including the precise nature of physicochemical interactions and the pharmacodynamic properties of both antimicrobials as well as concentrations and ratios at which the antimicrobials work in the most advantageous fashion can ultimately lead to more effective complementary therapeutic options. Such combination therapy may return even greater benefits by virtue of reducing the emergence of antimicrobial resistance through the administration of significantly lower levels of clinical antibiotics.

## 4. Materials and Methods

### 4.1. Bacterial Strains and Growth Conditions

The *S. agalactiae* strains were obtained from the Munster Technological University Culture Collection, having originally being isolated from blood culture and high vaginal swab samples from Cork University Hospital and University Hospital Limerick. The *S. capitis* strains (provided by Prof Frederick Laurent and Dr Marine Butin at Hospices Civils de Lyon, Lyon, France) were isolated from infected neonates (AV80, BA06, BD01) and two adult infections (AR18, AY41) from different geographical regions (Denmark, the Netherlands, Belgium, Germany and South Korea). *Lactococcus lactis* strains were grown at 30 °C in M17 broth (Oxoid, Waltham, MA, USA) supplemented with 0.5% glucose (GM17) or on GM17 agar plates containing 10 μg ml^−1^ chloramphenicol (Oxoid, Waltham, MA, USA). *L. lactis* transformants were grown on GM17 agar plates supplemented with nisaplin (Sigma, St. Louis, MO, USA)*. S. agalactiae* (*n* = 5) and *S. capitis* (*n* = 5) strains were grown in Tryptic Soy Broth (TSB) (Sigma, St. Louis, MO, USA) supplemented with 0.6% yeast extract (Sigma, St. Louis, MO, USA) (TSBYE) or TSBYE molten agar and incubated aerobically at 37 °C overnight. 

### 4.2. Whole Genome Sequencing of S. agalactiae Strains

Genomic DNA was extracted using the PureLink^TM^ Genomic DNA Kit (Invitrogen, Country of Origin) using the manufacturers guidelines for Gram positive bacteria. The DNA concentration of each sample was obtained using the Qubit DNA assay kit (Thermoscientific, Dublin, Ireland). Input DNA was prepared using the Rapid Barcoding Kit according to the manufacturer’s guidelines (Oxford Nanopore Technologies, Oxford, UK) prior to loading into the MinION (Oxford Nanopore Technologies).

### 4.3. Bioinformatic Analysis

Bioinformatic analysis was carried out on the genomes of the *S. agalactiae* (*n* = 5)*,* and *S. capitis* (*n* = 5) strains used in this study. ResFinder 4.1 (Center for Genomic Epidemiology) was used to identify acquired genes mediating antimicrobial resistance in the genomes of the bacteria. ORF Finder (NCBI) was used to search for nisin resistance proteins, NSR and NsrFP, and BLASTp (NCBI) was used to verify the predicted proteins.

### 4.4. Generation and Assessment of a Bank of Nisin Derivatives

Site-saturation mutagenesis of three codons, including threonine, lysine and methionine, at positions 2, 12 and 17 of the *nis*A gene, was carried out as described previously [28]. Briefly, pDF23 (pCI372-PV) was used as a template and oligonucleotides, NisT2deg-FOR and REV, NisK12deg-FOR and REV and Nis17deg-FOR and REV, containing an NNK codon potentially encoding for all twenty standard amino acids. Deferred antagonism agar-based assays were employed to assess the bioactivity of the nisin derivatives (PV, K12X-PV, T2X-PV, and M17X-PV) produced by each strain. Briefly, fresh overnight cultures of each *L. lactis* test culture was replicated on GM17/TSBYE agar plates containing 0.1% nisaplin using a 96-pin replicator (Boekel, Feasterville, PA, USA) and incubated overnight at 30 °C before being exposed to ultraviolet radiation for 45 min. The plates were then overlayed with GM17/TYSBYE agar (0.75% agar) and seeded (0.5% inoculum) with either *Lactococcus lactis* MG1614 (NSR-), the nisin resistance protein-expressing indicator strain *L. lactis* MG1614 pNP40 (NSR+) or TSBYE agar seeded with *Staphylococcus capitis* (NSR+) or *Streptococcus agalactiae* (NSR+) isolates. Plates were incubated overnight in appropriate conditions (37°C for *S. agalactiae*, *S. capitis* and 30 °C for *L. lactis* strains) and then examined for zones of inhibition. An enhancement in bioactivity was indicated by an increased zone of inhibition relative to that of the wild-type producer.

### 4.5. Identification of Nisin Derivatives

The changes to the nisA genes within the corresponding pDF23 (pCI372-PV) derivatives were established through DNA sequencing using the primer oDF101 5′-TCAGATCTTAGTCTTATAACTATACTG-3′ (Genewiz, Leipzig, Germany). Sequence alignments with the nisA gene were carried out with Lasergene Megalign 7.00 (DNAStar) to determine the nature of the codon changes.

### 4.6. Mass Spectrometry

Colony mass spectrometry of *L. lactis* transformants was performed as described previously [34]. Briefly, bacterial colonies were collected with sterile plastic loops and were mixed with 50 µL of 70% isopropanol (Fisher Scientific, Waltham, MA, USA). The suspensions were vortexed, and the cells were centrifuged at 14,000 rpm for 2 min. The supernatants were retained for analysis using a spectrometer (Shimadzu Biotech, Manchester, UK). Aliquots of 0.5 µL of matrix solution was placed onto the target and left for 1–2 mins before being removed. The residual solution was then air-dried, and the sample solutions were positioned onto the precoated sample spot. Following the addition of 0.5µL of matrix solution and air-drying, the samples were analysed in positive ion reflectron mode.

### 4.7. Nisin Purification

The nisin derivatives, PV, K12A-PV, T2A-PV, and M17A-PV were purified using a similar method to that described by Field [34]. Briefly, strains producing peptides of interest were inoculated (1% fresh overnight) into 2 L of modified Tryptone Yeast (TY) broth (Merck, Kenilworth, NJ, USA) supplemented with glucose (0.5% *v*/*v*) (Sigma-Aldrich, St. Louis, MO, USA) and β-glycerophosphate (2% *v*/*v*) (Sigma-Aldrich, St. Louis, MO, USA) and incubated at 30 °C for 16–18 h. The samples were centrifuged at 7000× *g* for 20 min and the supernatants were passed through a 60 g column of pre-equilibrated Amberlite XAD16N beads (Sigma-Aldrich, St. Louis, MO, USA). The beads were washed with 500 mL of 30% ethanol and then eluted with 500 mL of 70% isopropanol (IPA) (Fisher Scientific, Waltham, MA, USA) 0.1% trifluoroacetic acid (TFA) (Sigma-Aldrich, St. Louis, MO, USA). The cell pellets were resuspended in 300 mL of 70% IPA 0.1% TFA and stirred at room temperature for approximately 3 h. The cell debris from the cell pellets were removed by centrifugation at 5000× *g* for 10 min. These supernatants were combined with the previously eluted supernatants and then were evaporated using a rotary evaporator (Buchi, Flawil, Switzerland). The samples were adjusted to a pH of 4. To this 60 mL of methanol and 60 mL of water was passed through a 10 g (60 mL) Phenomenex SPE C-18 Column before the samples were applied to the column. The column was washed in 120 mL of 30% ethanol and the peptides were eluted in 80 mL of 70% IPA 0.1% TFA. Aliquots of 12 mL of nisin samples were concentrated to a volume of 2.5 mls and purified through HPLC using a Phenomenex (Phenomenex, Cheshire, UK) C12 Reverse-Phase (RP) HPLC column (Jupiter 4u proteo 90 Å, 250 mm × 10.0 mm, 4 µm) previously equilibrated with 25% acetonitrile—0.1% TFA. The column was subsequently developed in a gradient of 25% acetonitrile (Fisher Scientific, Waltham, MA, USA)—0.1% TFA to 50% acetonitrile containing 0.1% TFA from 10 to 40 min at a flow rate of 3.2 mL min–1. The relevant fractions were pooled, and acetonitrile was removed by rotary evaporation before the sample was freeze-dried (LABCONCO, Kansas City, MO, USA). Purified peptides were analysed using Axima MALDI-TOF Mass Spectrometry (Shimadzu Biotech, Manchester, UK) to confirm purity before use.

### 4.8. Minimum Inhibitory Concentration (MIC) Assay

MIC determinations were carried out in triplicate in 96-well microtiter plates (Sarstedt, Newton, NC, USA) as previously described [41]. Initially, 200µL of 1% bovine serum albumin (BSA) (Sigma-Aldrich, St. Louis, MO, USA) (in PBS) was added to the plates which were incubated at 37 °C for 30 min. The solution was removed, plates were washed with 200 µL PBS and then air dried in a laminar flow hood. Target strains (*Streptococcus agalactiae*, *Staphylococcus capitis* and *Lactococcus lactis*) were grown overnight in suitable media under appropriate conditions and were subsequently sub-cultured into fresh broth and allowed to grow to an optical density of ~0.5 (OD600). The culture was then diluted to a final concentration of 10^5^ CFU mL^−1^ in a volume of 1 mL. The peptides were resuspended in appropriate media to a stock concentration of 30 µL (*S. agalactiae and S. capitis* strains) and 10 µL (*L. lactis* strains). Nisin A and nisin derivatives were adjusted to a starting concentration of 15 µM (*S. agalactiae and S. capitis* strains) or 5 µM (*L. lactis* strains). Antibiotics were adjusted to starting concentrations of 50 µg/mL (Ampicillin and Penicillin), 100 µg/mL (Erythromycin and Vancomycin) and 200 µg/mL (Gentamicin). An aliquot of 100 µL of peptides and or antibiotics were added to the first well, mixed and then 2-fold serial dilutions of each peptide were made in TSBYE broth. Then, 100 µL of target strain was added to each test well, and the plates were incubated at 30 °C or 37 °C for 16 h. The MIC was read as the lowest peptide concentration causing inhibition of visible growth. Experiments were performed in triplicate.

### 4.9. Growth/Kill Curves

Growth curves were carried using methods described by Field et al. [41]. Concentrations used were established from MIC evaluation. 1/3 and 1/4 of the strain-specific MIC was used for the concentrations of nisin peptides within the range of 0.195 to 12.5 µg/mL and 1/3 and 1/4 of the MIC was used for the concentrations of antibiotics within the range of 0.0025 to 25 µg/mL. Briefly, overnight cultures of *S. agalactiae* (*n* = 5) and *S. capitis* isolates (*n* = 5) were diluted to a final concentration of $10^{7}$ CFU ${\mathrm{mL}}^{-1}$. Following this, 20µL of each culture was transferred into 1 mL of TSBYE broth supplemented with the relevant concentration of nisin wild type alone, nisin variants alone, antibiotics alone and peptide/antibiotic combinations. Subsequently, 0.2 mL of each treated culture was transferred to 96-well microtiter plates. Cell growth was monitored spectrophotometrically over 24 h periods at 37 °C using a spectrometer (Multiskan FC, Thermoscientific, Dublin, Ireland) at 600 nm. 

Kill curve assays were performed using methods described by Hayes et al. [31]. Briefly, fresh overnight cultures were diluted to a final concentration of $10^{5}$ CFU ${\mathrm{mL}}^{-1}$ and transferred into TSBYE broth supplemented with relevant concentrations of nisin wild-type or nisin variants alone (1.5 μg/mL for *S. agalactiae* and 12.5 μg/mL for *S. capitis*) and in combination with antibiotics (0.5 μg/mL of erythromycin and 6.25μg/mL of ampicillin) and incubated statically at 37 °C. During incubation, 100µL of each sample was taken at 0, 2, 4, 6 and 24 h. Cell growth was assessed by performing viable cell counts by diluting cultures in PBS solution and enumeration on TSBYE plates. Synergism was defined as ≥2 log_10_ CFU/mL reduction with the combination of antimicrobials relative to either antimicrobial alone after 24 h. A 1–2 log_10_ reduction in CFU/mL was defined as an additive effect and an increase or decrease of <1 log_10_ was defined as an indifferent effect. An antagonistic effect was defined as >2 log_10_ increase in CFU/mL after 24 h [28]. Experiments were performed in triplicate.

### 4.10. Statistical Analysis

Statistical analysis was carried out in R (R Core Team 2020). A normality test was used to evaluate if the data for each test was normally distributed. An independent Student *t*-test was used for normally distributed data. For data that was not normally distributed, Levene’s test of homogeneity was employed, followed by the independent Student *t*-test if equal variances were assumed, and the nonparametric Mann–Whitney test if equal variances were not assumed. The Kruskal–Wallis’ test was used to determine if there were any significant differences between the peptides alone and combination with antibiotics on the growth of the strains used in this study. Dunn’s test was used as a post hoc test for Kruskal–Wallis’ results with a significant difference between groups (*p* < 0.05). Statistical significance was defined as having a *p* value of <0.05.

## 5. Conclusions

*Streptococcus agalactiae* and the emerging pathogen *Staphylococcus capitis* are major aetiological agents of neonatal infections that often result in severe infections followed by long-lasting complications. Due to the rise of antimicrobial resistance and the lack of development of novel antibiotics, there is an urgent need for alternative treatment options to combat multi-drug resistant associated neonatal infections. Here, we report the screening of a large bank of bioengineered nisin derivatives and the in vitro synergy between conventionally used antibiotics and the bacteriocin, nisin and its variant, nisin PV against clinical neonatal isolates. This study demonstrates the potential of using nisin-antibiotic combination therapy to improve the efficacy of available antibiotics and, as a result, lower the rate of antibiotic resistance development. Although this study has revealed potentially promising synergistic interactions between nisin and antibiotics to more effectively target the neonatal pathogens *S. agalactiae* and *S. capitis*, it must be highlighted that in many instances these were antibiotic and strain dependent.

## Figures and Tables

**Table 1:** Resistance determinants identified in *S. agalactiae* and *S. capitis* isolates using bioinformatic analysis (ResFinder 4.1). Each isolate was examined for nisin resistant genes (*nsr* and *nsr*FP) as well as macrolide (*erm*B, *msr*D, *mef*A, *erm*A, *mre*A and *isa*C), tetracycline (*tet*M and *tet*O), β-lactam (*bla*Z, *mec*A), aminoglycoside (*aac*6′-*aph*2″), fusidic acid (*fus*B, fosfomycin *fos*D) and quarternary ammonium compound (*qac*A) resistance genes. The symbol “−“ represents the absence of the gene, and the symbol “+” represents the presence of the gene.

Bacterial Strains	Resistant Determinant																
	*nsr*	*nsr*FP	*msr*D	*mre*A	*mef*A	*mec*A	*tet*M	*tet*O	*erm*A	*erm*B	*erm*C	*Isa*C	*bla*Z	*acc*6′-*aph*2″	*fus*B	*qac*A	*fos*D
*S. agalactiae*																	
CIT 67	+	+	+	+	+	−	+	−	−	−	−	−	−	−	−	−	−
CIT 85	+	+	−	+	−	−	+	−	−	+	−	−	−	−	−	−	−
CIT 87	+	+	−	+	−	−	+	+	−	+	−	−	−	−	−	−	−
CIT 239	+	+	−	+	−	−	−	+	−	+	−	+	−	−	−	−	−
CIT 364	+	+	+	+	+	−	+	−	−	−	−	−	−	−	−	−	−
*S. capitis*																	
AR18	+	−	−	−	−	+	−	−	−	−	−	−	+	+	+	−	−
AV80	+	−	−	−	−	+	−	−	+	−	−	−	+	+	+	−	−
AY41	+	−	−	−	−	−	−	−	−	−	−	−	+	−	−	−	−
BA06	+	−	−	−	−	+	−	−	−	−	+	−	+	+	−	+	+
BD01	+	−	−	−	−	+	−	−	+	−	−	−	+	+	+	−	−

**Table 2:** Minimum inhibitory concentration (MIC) analysis of nisin peptides and antibiotics (penicillin, ampicillin, gentamicin, erythromycin, and vancomycin) against *S. agalactiae* and *S. capitis* isolates. Results are expressed as the average MIC obtained from three independent biological repeats. Values highlighted with an asterisk (*) indicates that the MIC obtained from the bioengineered peptide is decreased compared to the MIC obtained for the wild-type producer. Antimicrobials not tested are indicated by NT.

Strain	MIC (µg/mL)									
	Nisin					Antibiotics				
	Nisin A (WT)	PV	T2A-PV	K12A-PV	M17A-PV	Penicillin	Ampicillin	Gentamicin	Erythromycin	Vancomycin
*L. lactis* MG1614	0.02	0.06	0.26	0.13	0.56	NT	NT	NT	NT	NT
*L. lactis* *MG1614 pNP40*	0.26	0.03 *	0.13 *	0.06 *	0.06 *	NT	NT	NT	NT	NT
*S. agalactiae*										
CIT67	3	1.56 *	6.25	3	12.5	0.04	1.56	25	1	NT
CIT 85	6	12.5	25	12.5	NT	0.012	0.097	6.25	3.13	NT
CIT87	0.78	0.38 *	3	1.56	NT	0.02	0.78	25	0.39	NT
CIT239	12.5	6 *	25	25	NT	0.02	0.097	25	1.56	NT
CIT364	1.56	0.78 *	6.25	3	NT	0.02	0.78	25	0.78	NT
*S. capitis*										
AR18	25	25	NT	50	NT	>12.5	>12.5	>50	0.097	1.56
AV80	50	50	NT	50	NT	>12.5	>12.5	>50	12.5	3.13
AY41	25	6 *	12.5 *	50	12.5	0.39	6.25	0.39	0.195	1.56
BA06	12.5	6 *	NT	100	NT	12.5	>12.5	>50	12.5	1.56
BD01	25	25	NT	100	NT	6.25	>12.5	>50	6.25	3.13

## Data Availability

The data presented in this study are available on request from the corresponding author.
